# The CLCF1-CNTFR axis drives an immunosuppressive tumor microenvironment and blockade enhances the effects of established cancer therapies

**DOI:** 10.21203/rs.3.rs-4046823/v1

**Published:** 2024-03-22

**Authors:** Eric Sweet-Cordero, Kieren Marini, Emma Champion, Alex Lee, Isabelle Young, Stanley Leung, Nicolas Mathey-Andrews, Tyler Jacks, Peter Jackson, Jennifer Cochran

**Affiliations:** University of California System; Division of Oncology, Department of Pediatrics, University of California San Francisco; Division of Oncology, Department of Pediatrics, University of California San Francisco; University of California, San Francisco; Division of Oncology, Department of Pediatrics, University of California San Francisco; Division of Oncology, Department of Pediatrics, University of California San Francisco; Massachussets Institute of Technology; David H. Koch Institute for Integrative Cancer Research; Stanford University School of Medicine; Stanford University

## Abstract

Tumors comprise a complex ecosystem consisting of many cell types that communicate through secreted factors. Targeting these intercellular signaling networks remains an important challenge in cancer research. Cardiotrophin-like cytokine factor 1 (CLCF1) is an interleukin-6 (IL-6) family member secreted by cancer-associated fibroblasts (CAFs) that binds to ciliary neurotrophic factor receptor (CNTFR), promoting tumor growth in lung and liver cancer^1,2^. A high-affinity soluble receptor (eCNTFR-Fc) that sequesters CLCF1 has anti-oncogenic effects^3^. However, the role of CLCF1 in mediating cell-cell interactions in cancer has remained unclear. We demonstrate that eCNTFR–Fc has widespread effects on both tumor cells and the tumor microenvironment and can sensitize cancer cells to KRAS inhibitors or immune checkpoint blockade. After three weeks of treatment with eCNTFR-Fc, there is a shift from an immunosuppressive to an immunostimulatory macrophage phenotype as well as an increase in activated T, NKT, and NK cells. Combination of eCNTFR-Fc and αPD1 was significantly more effective than single-agent therapy in a syngeneic allograft model, and eCNTFR-Fc sensitizes tumor cells to αPD1 in a non-responsive GEM model of lung adenocarcinoma. These data suggest that combining eCNTFR-Fc with KRAS inhibition or with αPD1 is a novel therapeutic strategy for lung cancer and potentially other cancers in which these therapies have been used but to date with only modest effect. Overall, we demonstrate the potential of cancer therapies that target cytokines to alter the immune microenvironment.

## Introduction

Lung cancer remains one of the most common cancers and there is a need for approaches to enhance currently available therapies. While inhibitors of KRAS G12C have shown efficacy in multiple clinical trials, resistance mechanisms rapidly emerge^4^. Similarly, while immune checkpoint inhibitors (ICIs) are effective in some patients, many do not respond^5,6^. The tumor microenvironment (TME) supports tumor growth via paracrine signaling from cancer-associated fibroblasts (CAF)^7^. Dysregulation of cytokines and other secreted factors is a universal feature in cancer, yet strategies to use this knowledge for therapeutic benefit have had limited impact^8^. Cytokine-directed therapies could be leveraged to enhance the effects of existing therapies by modulating the TME.

CLCF1 (NNT-1/BSF-3) is a member of the IL-6 family of cytokines and is highly expressed in lung and pancreatic adenocarcinomas, as well as other tumor types (**Extended Data** Fig. 1) and was initially identified in T cell lymphomas^9^. The CLCF1 receptor, CNTFR, forms a trimeric complex with leukemia inhibitory factor receptor (LIFR) and gp130^10^. CLCF1 binding to this tripartite receptor complex triggers downstream signaling cascades mediated by JAK-STAT, MAPK, and other pathways^11^, driving tumorigenesis. We previously described a mechanism by which secretion of CLCF1 by CAFs promotes tumor growth in mouse models of lung adenocarcinoma^1^.

eCNTFR-Fc is a soluble ligand trap that binds and sequesters CLCF1^3^. eCNTFR-Fc inhibits tumor growth as a single agent in xenograft and GEM models. Blockade of CLCF1 by eCNTFR-Fc has intrinsic effects on tumor cell signaling via STAT3 and Erk phosphorylation, leading to apoptosis and decreased proliferation in mouse tumor models^3^. Here, we dissect both the cell autonomous and non-cell autonomous effect of CLCF1 signaling in lung cancer using eCNTFR-Fc. Using single-cell and in situ spatial analysis, we demonstrate that CLCF1 plays a major role in driving an immunosuppressive TME and that combination of CLFCF1 inhibition with established cancer therapies can improve efficacy in NSCLC. These results establish a new therapeutic paradigm potentially widely relevant across many tumor types.

## Results

### Cell autonomous single agent eCNTFR-Fc treatment results in suppression of the RAS/MAPK signaling axis.

**Figure 1:**
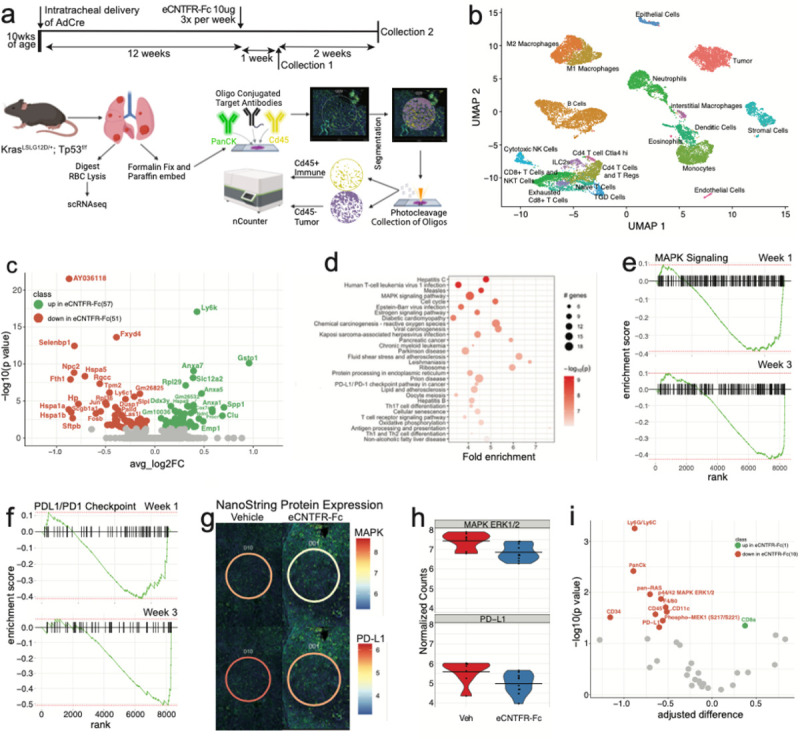
Tumor cell intrinsic mechanisms of eCNTFR-Fc. **a.** Schematic of the timeline and processing for sample treatment, collection and processing for single cell and spatial transcriptomics. Samples were collected at one- and three-weeks post treatment start. **b**. UMAP visualization of 20 identified cluster from 19189 cells profiled by 3’scRNA analysis. **c.** Differential gene expression analysis of tumor cells at 3 weeks post treatment with eCNTFR-Fc, green is up in eCNTFR-Fc treated and orange is down in eCNTFR-Fc treated. **d.** Pathway analysis of differentially expressed genes in the tumor cluster. **e-f.** GSEA enrichment plots of selected pathways. **e.** MAPK GSEA analysis. **f.** PD-L1/PD-1 Checkpoint in Cancer GSEA analysis. **g-h.** Protein profiling of matched vehicle and eCNTFR-Fc treated lung tumors using Nanostring. Lung sections were imaged using three-color fluorescence of CD45 (yellow), PanCK (green) and DNA (blue) to establish overall tissue morphology. **g.** Examples of regions from Vehicle (left) and eCNTFR-Fc (right) treated tissues, where line designating the ROI corresponds to expression of the target protein. p42/44 MAPK (top) and PD-L1 (bottom) protein. **h.** Violin plots of protein expression for p42/44 MAPK (top) and PD-L1 (bottom). **i.** Differential protein analysis of tumor regions at 3 weeks post treatment with eCNTFR-Fc where green is up in eCNTFR-Fc treated and orange is down in eCNTFR-Fc treated.

To assess the effect of eCNTFR-Fc on tumor development, we used an autochthonous, highly aggressive GEM model of lung adenocarcinoma^12,13^. Intratracheal instillation of adenovirus (Ad5-CMV-Cre) in *Kras*^G12D−LSL^/*Trp53*^f/f^ (KP) mice led to consistent development of adenocarcinoma. Tumors were induced in the lungs of KP mice and treated with vehicle or eCNTFR-Fc. Mice were sacrificed at one- or three-weeks after treatment initiation and lung cells harvested for analysis (Fig. 1a). To understand the effects of eCNTFR-Fc on tumorigenesis, treated tumors were analyzed using single-cell RNAseq and spatial transcriptomics. scRNAseq analysis was performed on 24,783 cells across all treatments. Cell types were identified based on reference cell annotations and manual curation (Fig. 1b **and Extended Data** Fig. 2a). Single cell transcriptome analysis of the tumor compartment identified effects of eCNTFR-Fc on genes related to immune modulation at 3 weeks (Fig. 1c). For example, eCNTFR-Fc upregulated *Gst01*, a gene coding for Glutathione transferase Omega 1, a protein previously shown to play a pro-inflammatory role^14^. eCNTFR-Fc also downregulated SelenBP1 expression which is associated with immune infiltration and is negatively correlated with the presence of NK cells, T helper cells, central and effector memory T cells and CD8 + T cells^15^. Pathway analysis showed enrichment of pathways related to immune function including antigen processing and presentation, viral infection and T cell receptor signaling (Fig. 1d). In contrast, the MAPK and PD-L1/PD1 immune checkpoint pathways were downregulated in tumor cells as shown by GSEA enrichment analysis (Fig. 1e–f and **Extended Data** Fig. 2b-c) at both 1- and 3-weeks post treatment. Downregulation of MAPK is consistent with prior data indicating that eCNTFR-Fc inhibits KRAS signaling^3^. PD-L1 signaling is possibly reduced due to decreased expression of *Jun, Hif-1* a, and *Nfkb* in eCNTFR-Fc treated cells, all known regulators of PD-L1 expression^16,17^. Overall, eCNTFR-Fc treatment resulted in consistent dysregulation of 108 genes in the tumor cell compartment in the *Kras*^G12D^/*Trp53*^f/f^ model.

To further assess the consequences of eCNTFR-Fc on tumor cells, spatial analysis of changes in protein expression was performed on the Nanostring DSP Platform. Lung sections were stained for CD45 (yellow), PanCK (green), and DNA (blue) to establish overall tissue morphology. Regions of interest (ROIs) of 300 μm in diameter were selected for molecular profiling with a 43-plex oligonucleotide–antibody cocktail designed to query the MAPK pathway and key immune subtypes (see methods). Examples of the lung morphologies are shown in **Extended Data** Fig. 3. Protein expression analysis in situ between vehicle and eCNTFR-Fc treated tumor cells also identified decreases in p42/44 MAPK and PD-L1 (Fig. 1g–h). Differential protein expression analysis demonstrated decreases in total RAS, MEK, and ERK expression (Fig. 1i), which aligns with the decreases in gene expression seen using single cell transcriptome analysis.

RAS/MAPK pathway alteration in response to inhibition of the CLCF1/CNTFR signaling axis was also seen in human lung cancer cell lines (**Extended Data** Fig. 4). Human LUAD cell lines with stably integrated Cas9 were transfected with sgRNA guides to Control or CNTFR (Synthego) to generate Control (sgNT) or CNTFR knockout cell lines. Three KRAS mutant lines, (A549 (G12S), H23 (G12C), H358 (G12C)), and KRAS wildtype lines (H1437 and H1975) were evaluated. As previously reported, cell lines with KRAS mutations were generally more sensitive to KRAS knockout/knockdown^18^ and KRAS mutant cell lines were more susceptible to eCNTFR treatment^3^. An exception was the KRAS G12C mutant cell line, H23, which did not show sensitivity to KRAS knockout (**Extended Data** Fig. 4a) consistent with previous reports^18–21^. The two most KRAS-dependent cell lines, determined by either knockout or with RNA interference, A549 and H358, were the most affected by CNTFR knockout (**Extended Data** Fig. 4b). Individual growth curves for A549, H358 and H23 with control or CNTFR knockouts conditions are shown in **Extended Data** Fig. 4c-e. Cells with a knockout of CNTFR had a lower capacity to form colonies compared to the non-targeting guides in A549 and H358, but not H23 (**Extended Data** Fig. 4f-h), consistent with the growth assay results.

To further define the tumor-intrinsic effects of CLCF1-CNTFR signaling, kinase signaling was analyzed under serum-starved and serum-stimulated conditions in sgNT and sgCNTFR A549 clones. CNTFR knockout resulted in abrogation of the JAK/STAT pathway and partial inhibition of the MEK/ERK and AKT pathways (**Extended Data Fig**. 5a). Western blotting also confirmed changes to the kinetics and intensity of ERK phosphorylation (**Extended Data Fig. 5b-e**). The effects on ERK signaling were not observed in H23 cells, which, as noted above, are refractory to KRAS inhibition (**Extended Data Fig**. 5f-g). In KRAS mutant (G12S) LUAD cell line A549, CNTFR knockout led to complete abrogation of the JAK/STAT and partial inhibition of the MEK/ERK and AKT pathways (**Extended Data Fig. 5b-c**).

While H23 cells were not sensitive to either KRAS inhibition or CNTFR knockout alone, knockout of CNTFR increased sensitivity to KRAS G12C inhibition in H23 and H1792 knockout cell lines (**Extended Data Fig. 6a-b**). The same effect was observed when WT H23 cells were treated with eCNTFR-Fc in combination with KRAS G12C inhibition (**Extended Data Fig. 6c**). When KRAS G12C inhibition was combined with CNTFR knockout, there was an almost complete abrogation of p-ERK, indicating a synergistic effect on inhibition of MAPK signaling (**Extended Data Fig. 6d-e**). Overall, these results indicate that the CLCF1-CNTFR axis supports proliferation in the presence of KRAS inhibition and that abrogation of this axis using eCNTFR-Fc is a potential therapeutic strategy relevant to human cancers that carry KRAS mutations.

### Blockade of CLCF1-CNTFR signaling remodels the tumor microenvironment.

**Figure 2:**
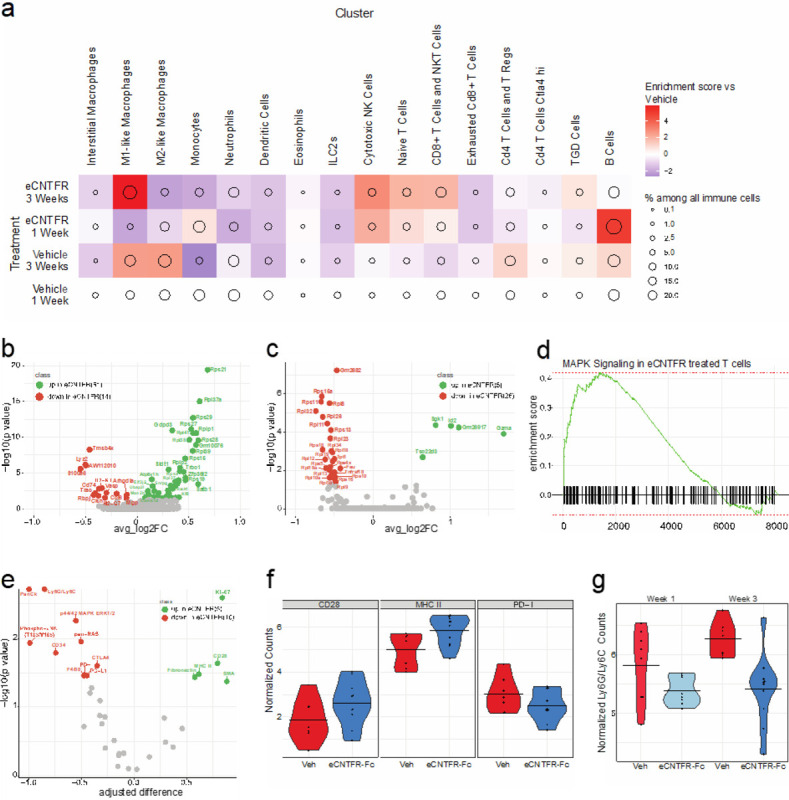
eCNTFR-Fc alters the immune microenvironment in lung adenocarcinoma. **a.** Tumors were treated with either vehicle or eCNTFR-Fc for 1- or 3-weeks. Plot shows relative enrichment for immune cells where circle size is % among all immune cells and color is enrichment compared to vehicle treated cells. **b-c.** Differential gene expression analysis of the effector immune cell populations after treatment with eCNTFR-Fc. **b.** CD8+ T cells and NKT cells population after one week treatment with eCNTFR-Fc. **c.** CD8+ T cells and NKT cells population after three weeks treatment with eCNTFR-Fc. **d.** GSEA analysis of the MAPK Pathway effector T cells at the 3-week timepoint. **e.** Differential protein analysis of immune regions at 3 weeks post treatment with eCNTFR-Fc where green is up in eCNTFR-Fc treated and orange is down in eCNTFR-Fc treated. **f.** Violin plots of protein expression for CD28, MHC II and PD-1. **g.** Violin plots of LY6G/LY6C expression at one- and three-weeks post eCNTFR-Fc treatment.

Single cell transcriptome analysis identified significant changes to immune cell populations in response to eCNTFR-Fc treatment. After one week of treatment with eCNTFR-Fc, macrophages were significantly depleted, whereas both B and T cells expanded (Fig. 2a). After three weeks of treatment, these changes were more apparent, with a strong enrichment of M1-like (Cd11c^+^Cd206^lo^Cd86^hi^) macrophages and additional depletion of M2-like (Cd11c^+^Cd206^hi^) macrophages. At both treatment timepoints, there were fewer CD8 + T cells with exhaustion markers (Lag3, Ctla4, Pd-1, Tigit, and Havcr2) and an increase in cytotoxic CD8 + T cells, NKT cells, and NK cells.

The effect of eCNTFR-Fc treatment on T cells was further evaluated using differential gene expression analysis of both naïve and CD8 + subsets (Fig. 2b–c). After one week of eCNTFR-Fc treatment, there was a significant upregulation of ribosomal protein-coding genes (Fig. 2b), which is known to occur prior to T cell expansion^22^, consistent with the observed enrichment of mature effector T cell populations at the three-week timepoint. Differential gene expression analysis of eCNTFR-Fc treated CD8 + T cells and NKT cells at the three-week timepoint identified downregulation of these ribosomal proteins and upregulation of expression of *Id2* and *Gzma* (Granzyme A)^22^, suggesting these cell populations have passed the expansion phase and have reached a mature cytotoxic phenotype (Fig. 2c). In parallel, MAPK signaling increased in T cells after eCNTFR-Fc treatment, in contrast to the observed effect in the tumor cell population and suggesting that this is an indirect effect.

Spatial analysis was used to analyze proteins associated with CLCF1-dependent changes in the immune microenvironment. After three weeks of treatment with eCNTFR-Fc, the T cell activation markers CD28 and MHC II were upregulated, whereas the T cell exhaustion markers CTLA4, PDL1, and PD-1 were downregulated^23,24^ (Fig. 2e–f). CD28 is required for PD-1 blockade to efficiently kill cancer cells^25^. Ly6C/G expression was also decreased in CD45 + cells at both eCNTFR-Fc treatment timepoints (Fig. 2g). The Ly6 antibody has dual specificity, binding to both Ly6C and Ly6G, which marks two separate populations. Ly6C marks a subset of monocytes/macrophages, and Ly6G marks neutrophils^26–28^, thus these two populations cannot be distinguished using this marker. The single cell analysis corroborates losses in both populations, with greater losses seen in the neutrophil populations when comparing vehicle to eCNTFR-Fc (Fig. 2a). Neutrophils have been shown to contribute to the immunosuppressive environment and facilitate immune evasion, reducing the effectiveness of immune checkpoint inhibition^29^. Taken together, eCNTFR-Fc treatment leads to an immune phenotype consistent with decreased immunosuppression and enrichment of activated effector immune cells.

### Blockade of CLCF1 signaling potentiates the effect of checkpoint blockade.

**Figure 3:**
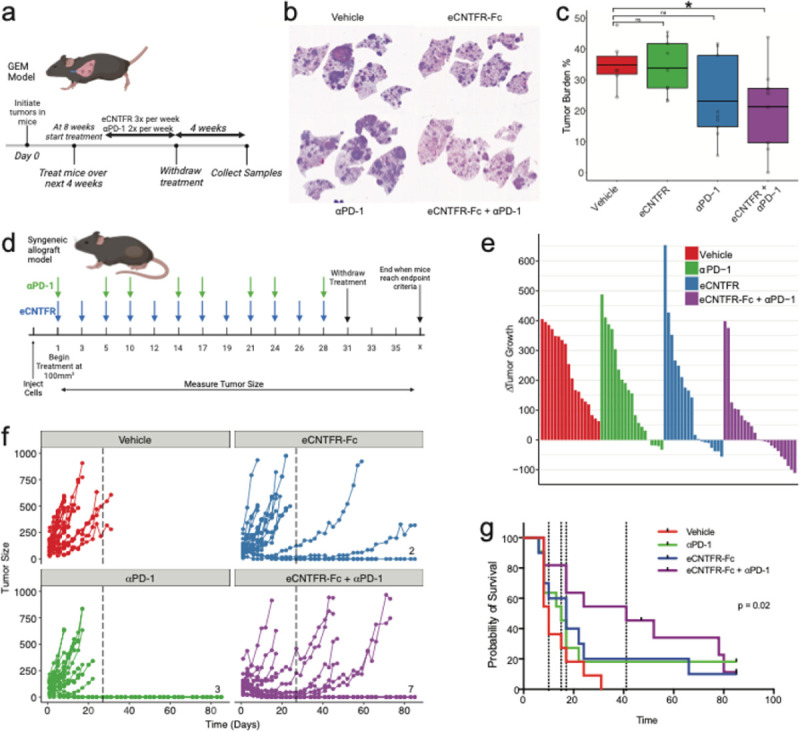
Combination of eCNTFR-Fc and αPD-1. **a-c.** Treating GEM model of LUAD with vehicle, eCNTFR-Fc alone, αPD-1 alone or eCNTFR-Fc and αPD-1 in combination, n = 6 for vehicle and n = 9 treated groups**. a.**Schematic of the timeline of initiation and treatment schedule of KP GEM model. **b.** Representative H&E images of lungs from mice treated with vehicle, eCNTFR-Fc alone, αPD-1 alone or eCNTFR-Fc and αPD-1 in combination. **c.** Effect of treatment on tumor burden %, p < 0.05 using Wilcoxon signed rank sum test. **d-g.**Syngeneic allograft model of LUAD treated with vehicle, eCNTFR-Fc alone, αPD-1 alone or eCNTFR-Fc and αPD-1 in combination, n ≥ 18. **d.**Schematic of the treatment schedule for dosing of eCNTFR-Fc and αPD-1. **e.** Waterfall plot of change in tumor growth, per tumor, one week after start of treatment. **f.** Spider plots of tumor growth for individual tumors. **g.** Kaplan-meier curve of survival for vehicle, single agent, and combination therapy, p = 0.02 using Log-rank test.

The changes to the immune compartment after eCNTFR-Fc treatment suggest that the blockade of CLCF1 signaling could potentiate the effect of checkpoint inhibitor therapy in lung cancer. To test this, we tested the efficacy of combined therapy with eCNTFR-Fc and αPD-1 in the KP GEM model. At 8-weeks post tumor initiation, mice were treated with vehicle, eCNTFR-Fc alone, αPD-1 alone, or a combination of eCNTFR-Fc and αPD-1 for 28 days, and tumors were collected at 16 weeks (Fig. 3a). A representative image of lungs under each of the different treatments is shown in Fig. 3b. Mice treated with single agent eCNTFR-Fc demonstrated decreased tumor burden compared to vehicle-treated controls, while single agent αPD-1 had little effect (Fig. 3c). The effect of eCNTFR alone was less significant than in prior work^3^, most likely due to the higher tumor burden in mice treated in this study (see methods). Strikingly, combination of eCNTFR-Fc and αPD-1 led to a greater than additive effect and significantly decreased tumor burden when compared to vehicle, suggesting that eCNTFR-Fc is sufficient to overcome the poor T cell responses, which usually renders immunotherapy ineffective in the KP lung adenocarcinoma model^30–35^.

To further dissect this drug interaction, we used a syngeneic allograft model where mouse LUAD cells, harvested from a GEM model mouse, were injected and grew subcutaneously in the mouse flank. Mice were implanted and treated with vehicle, eCNTFR-Fc alone, αPD-1 alone, or a combination of eCNTFR-Fc and αPD-1 for up to 28 days, and tumors were collected once mice reached endpoint (Fig. 3d). While eCNTFR-Fc or αPD-1 alone did have an effect on tumor growth in this model, with 7 of 18 tumors having decreased tumor volume and 2 achieving a complete response to eCNTFR-Fc alone and 4 of 19 tumors out of having decreased tumor volume and 3 achieving a complete response to αPD-1 alone. Mice that received a combination of eCNTFR-Fc and αPD-1 had significantly decreased tumor growth (Fig. 3e–f). 11 of 22 mice had tumor regression, with 7 mice achieving a complete response, and the remaining mice exhibiting more intermediate responses (Fig. 3e–f). Combination treatment also significantly increased the survival of mice in comparison to the vehicle and single agent treated mice (Fig. 3g).

### Composition of the immune system is altered after combination therapy with eCNTFR and αPD-1.

**Figure 4:**
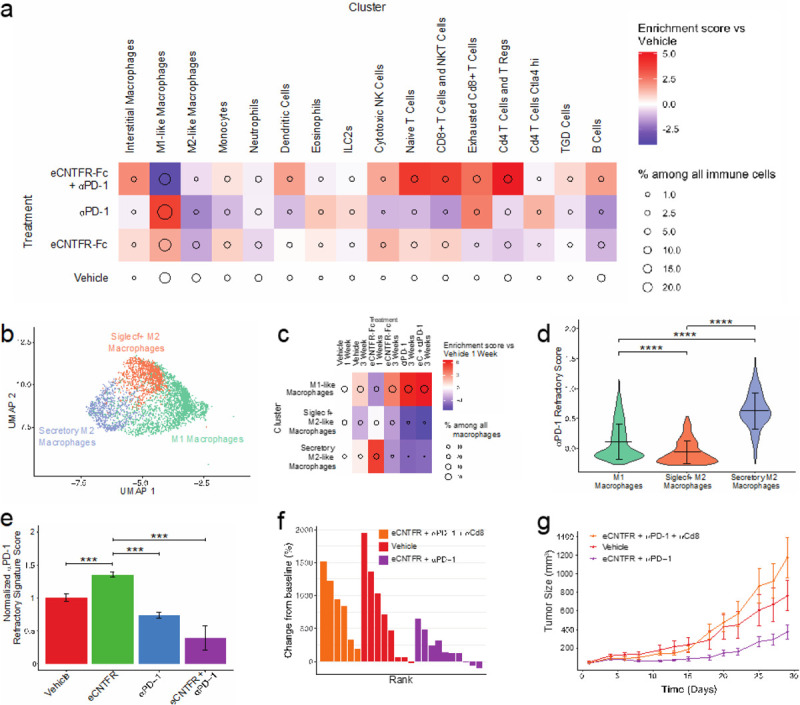
Effects of eCNTFR-Fc and αPD-1 combination therapy on the immune composition of tumors. **a.** Tumors were treated for 3-weeks with vehicle, eCNTFR-Fc alone, αPD-1 alone or eCNTFR-Fc and αPD-1combination and subpopulations of cells were analyzed using scRNAseq. Plot shows relative enrichment for immune cells where circle size is % among all immune cells and color is enrichment compared to vehicle treated cells. **b.** UMAP of 3 clusters identified from 4452 macrophages, clustered at higher resolution. **c.** Relative enrichment plot for the three macrophage clusters where circle size is % among all alveolar macrophages and color is enrichment compared to vehicle treated condition. **d.** Violin plot of the module score for refractory to PD-1 gene signature for each of the different macrophage populations **** = p < 0.0001. **e.** Bar plot of the module score for NSCLC refractory to PD-1 gene signature for the tumor population after treatment with vehicle, eCNTFR-Fc alone, αPD-1 alone or eCNTFR-Fc and αPD-1 in combination, *** = p < 0.001. **f-g.** Treatment of LUAD syngeneic allograft model with vehicle, eCNTFR-Fc + αPD-1 or eCNTFR-Fc and αPD-1 in combination with αCD8, n ≥ 6 per group. **f.** Waterfall plot of change in tumor growth. **g.** Growth curve of syngeneic allograft after treatment.

To evaluate the mechanistic basis for the enhanced response to αPD-1 when combined with eCNTFR-Fc, we performed single cell sequencing of lung tumors treated with single or combination therapy (Fig. 4a). Treatment with αPD-1 alone resulted in the depletion of Cd206^hi^ M2-like macrophages and an increase of Cd86^hi^Cd206^lo^ M1-like macrophages, similar to eCNTFR-Fc, but without the reciprocal enrichment of T cell effector populations. Cytotoxic NK, NKT, Cd8 + T cells and naïve T cells all were decreased after treatment with αPD-1. Notably, the only enrichment in T-cell populations after αPD-1 was an increase in exhausted CD8 + T cells, which can no longer mount an effective anti-tumor response. In contrast, when eCNTFR-Fc was combined with αPD-1, there was an overall decrease in the relative number of macrophages. As with single agent eCNTFR-Fc treatment, there was enrichment in cytotoxic CD8 + T cells, NKT cells, and NK cells, but additionally, there were increases in CD4 + T cells and T Regs (marked by Cd4 and Foxp3, respectively). Interestingly, T Regs have classically been shown to have decreased anti-tumor responses^36^; however, some newer studies suggest careful control of CD4 expressing cells, including T Regs, are required for an optimal immune environment to promote anti-tumor efficacy of immunotherapy^37^.

Subsetting of the macrophage population allowed for further characterization of these cells and increased resolution, allowing for the identification of 3 macrophage subpopulations (Fig. 4b): one M1-like (immunostimulatory) and two M2-like (immunosuppressive) populations. Treatment with eCNTFR-Fc resulted in the depletion of CD206 + M2-like macrophages, similar to single agent αPD-1 treatment (Fig. 4c). We observed expression of secreted cytokines in one subset of the M2-like macrophages. Expression of a set of cytokines has been correlated to low response to immunotherapy in NSCLC^38,39^. Using this “refractory to PD-1” gene signature, we then calculated a score for the expression of the gene signature on a per-cell basis and identified an M2-like subset with a high PD-1 treatment refractory signature score (Fig. 4d), suggesting that these cells may partially mediate response to αPD-1. Additionally, it may suggest that eCNTFR-Fc contributes to the sensitization to αPD-1 by preventing the differentiation of naïve monocytes into this subset of macrophages, preventing subsequent secretion of refractory cytokines.

Although αPD-1 treatment alone is sufficient to show the depletion of M2-like macrophages, this does not translate to the sensitivity to αPD-1 in the GEM model. This is likely due to the lack of reciprocal activation of effector cell populations and the possibility of secretion of refractory cytokines by multiple cell types (**Extended Data Fig. 7**), including other myeloid populations, monocytes, DCs and neutrophils, stromal cells, and tumor cells themselves. When looking at the response of tumor cells to treatment, tumor cells treated with αPD-1 alone showed increased expression of the genes associated with PD-1 refractory signature responses (Fig. 4e). However, treatment with eCNTFR-Fc alone or in combination with αPD-1 led to significant loss of the refractory signature, suggesting that suppression of the CLCF1/CNTFR signaling axis is preventing the release of these cytokines. This is not overly surprising as several genes in the signature are members of the IL6 subfamily or are targets of STAT3, which are canonically activated downstream components of CLCF1/CNTFR signaling^40,41^. To further confirm whether tumor cells are contributing to the cytokine expression changes in response to CLCF1/CNTFR signaling, we evaluated cytokine expression under serum-starved and serum-stimulated conditions in A549 control and CNTFR knockout cell lines using a cytokine array. CNTFR knockout led to decreased expression of IL-6 and IL-11 under both serum-starved and stimulated conditions (**Extended Data Fig. 8).** VEGF^42^ had decreased expression in CNTFR knockout cells and VEGF expression did not respond as well to serum stimulation, consistent with the observed decrease in STAT3 activation. VEGF has also been shown to directly influence T cell response by increasing the expression of immunosuppressive checkpoints^43,44^. Similarly, we saw decreased expression of Angiopoietin-2, previously described as a biomarker of poor response to immunotherapy^45^ and linked to poor prognosis for patients with NSCLC^46^.

To evaluate whether this response to combination treatment requires T cell activation, tumors were treated with a CD8 antibody to deplete CD8 + T cells in conjunction with the eCNTFR-Fc and αPD-1 combination therapy. CD8 + T cell depletion completely abolished the effect of the eCNTFR-Fc and αPD-1 combination (Fig. 4f–g), indicating that this effect is T-cell dependent. In summary, the combination of eCNTFR-Fc and αPD-1 treatment alters the cellular composition of the TME, leading to a phenotype of decreased immunosuppression and increased enrichment of effector immune cells and T cell mediated anti-tumor activity.

## Discussion

We demonstrate a previously uncharacterized role for the CLCF1-CNTFR signaling in maintaining the pro-tumorigenic TME of lung cancer. The blockade of this signaling pathway by eCNTFR-Fc has anti-oncogenic effects through two distinct mechanisms. First, cell lines and tumors sensitive to CNTFR knockout or inhibition show consistent changes to the KRAS/MAPK pathway. Additionally, KRAS mutant LUAD cell lines insensitive to KRAS inhibition become sensitized when CNTFR is knocked-out or treated with eCNTFR-Fc. These findings support the hypothesis that CLCF1 potentiates oncogenic KRAS signaling *in vivo* and provides preclinical support for the use of eCNTFR-Fc in combination with the emerging class of direct KRAS inhibitors to treat KRAS mutant LUAD.

Single cell and spatial data indicate that eCNTFR-Fc broadly alters the TME from an immune-suppressive, tumor-promoting environment, towards a more immune-stimulatory, tumor-inhibitory phenotype. After treatment, we see decreased enrichment of immunosuppressive tumor-associated neutrophils and macrophages with increases in enrichment of effector T cells and NK cells. Combining eCNTFR-Fc with αPD-1 led to a significant increase in recruitment of cytotoxic NK, NKT and CD8 + T cells to tumors in a genetically engineered model of lung cancer that has been demonstrated to reproduce key features of the human disease. Treatment with eCNTFR-Fc sensitizes tumors to αPD-1 treatment in a highly aggressive GEM model, providing strong preclinical support for clinical trials of this combination. These results point to the potential increased efficacy of checkpoint inhibitor therapy when combined with agents such as eCNTFR that can reprogram the tumor microenvironment towards a more anti-oncogenic phenotype.

In hepatocellular carcinoma, CLCF1 affects the expression of CXCL6 and TGFb, promoting neutrophil recruitment and polarization of tumor-associated neutrophils towards the N2 phenotype^2^. These tumor-associated neutrophils, in turn, recruited tumor-associated macrophages, which promoted resistance to Sorafenib^47^. CLCF1 was also shown to contribute directly to Sorafenib resistance through PI3K/AKT signaling^48^. In glioblastoma (GBM), another IL6 family member, LIF, has also been shown to epigenetically silence CXCL9 in macrophages. Blockade of LIF removed silencing of CXCL9 expression, which promoted T cell migration and sensitization of GBM to αPD-1 therapy^49^. We previously established a LIFR decoy receptor for the treatment of pancreatic ductal adenocarcinoma^50^ and, based on our results with eCNTFR-Fc, suggest targeting LIF in the context of αPD-1 combination therapy, which may also be of therapeutic benefit in LUAD.

Over the last several decades, there has been widespread interest in the idea of inhibiting cytokines and cytokine receptors as an anti-oncogenic strategy. Interleukins of the IL6 subfamily have been among the best studied^51^ since the IL6-JAK-STAT signaling pathway is frequently altered in cancer and autoimmune diseases^52^. However, relatively little attention has been centered on the anti-oncogenic effects of CLCF1 specifically. The results described here demonstrate the untapped potential of targeting the complex signaling networks between cells in the tumor microenvironment as a strategy to potentiate cancer therapy.

## Methods

### Ethics Statement.

Mice were maintained and animal experiments performed in accordance with policies approved by the Institutional Animal Care and Use Committee at the University of California San Francisco (AN197629–00C).

### Mice.

#### Genetically engineered mouse model.

Lox-stop-Lox-Kras^G12D^ (129Sv/Jae)^4553^, Trp53^fl/fl^(FVB)^4654^, (C57BL/6J) mice were maintained in a virus-free environment. Mice were intratracheally infected with 1×10^7^ p.f.u. of adenovirus-expressing Cre (University of Iowa) between ages 10–12 weeks using a mouse intubation pack (Hallowell EMC)^4553^. Briefly, mice were administered a mixture of Ketamine (8 mg/ml, KETASET, Zoetis) and Xylazine (1.6 mg/ml, no. X1251–5G, Sigma). Mice were then placed on their back and an otoscope was placed in their mouth to visualize the vocal cords, then a cannula and guide wire were used to insert the cannula into the trachea of the mouse. The guide wire was removed, and virus added to the cannula to be aspirated by the mouse. Mice were dosed with eCNTFR–Fc (10 mg kg^−1^) or PBS (vehicle) by i.p. injection for 4 weeks, three times per week, beginning as indicated by the experiment. Mice were weighed at the beginning of study and periodically throughout drug treatment. Equal number of male and female mice were utilized. Lungs from tumor-bearing mice were collected and processed as indicated.

#### Tumor burden analysis.

Aiforia histological quantification of mouse lung tumor burden was performed by an automated deep neural network, developed by Aiforia Technologies^55^. Briefly, a trained convolutional neural network (CNN) for semantic multi-class segmentation was used to classify and detect lung parenchyma and NSCLC tumors.

#### Syngeneic mouse model.

Mice were subcutaneously injected into the flanks at about 10 weeks of age with 5.0 × 10^5^ cells in 30 μL of Matrigel. Once tumors reached an average of 100 mm3, mice were randomized into treatment groups. Mice were dosed with eCNTFR-Fc (10 mg kg^−1^), anti-PD-1 (5 mg kg^−1^, clone 29F1.A12, BioXCell) or PBS (vehicle) by i.p. injection for 4 weeks. Tumors were measured three times weekly. Mice were euthanized at standard humane endpoints including when tumors reached 1000 mm^3^.

### Single Cell Experiment.

Tracheas were exposed and the lungs were perfused with phosphate buffered saline (PBS). Whole lungs were removed, and tumors were macrodissected and minced with razor. Samples were then digested with Dulbecco’s Modified Eagle Medium/F12 (no. 11965–092, Gibco) containing 2 mg/mL collagenase/Dispase (no. 11097113001, Roche Applied Science) and 0.025 mg/mL DNAse (no. AM2238, Invitrogen) at 37°C for one hour with agitation. Cells were filtered through 70 μm strainers. Red blood cells were lysed with hypotonic buffer (15mM NH4Cl, 10mM KHCO3 and 0.1mM EDTA) for one to two minutes, then washed with Dulbecco’s Modified Eagle Medium/F12 supplemented with 10% fetal bovine serum (no. 35–011-CV, Corning). Dead cells were removed using the MACS Dead Cell Removal Kit (no. 130-090-101, Miltenyi Biotec) as per manufacturer’s instructions. Cells were counted using a Countess II. 17,000 cells were loaded onto the Chromium Next GEM Chip G. Library preparation was done as per 10x Genomics instructions and samples were sequenced on an Illumina NovaSeq 6000.

Transcripts were aligned to mouse (GRCh38) and a feature-barcode count matrix was generated using Cell Ranger (4.02). This was imported into R using the Seurat package (4.1.0) for downstream analysis^56^. Expression data was then tagged by experiment, scaled and cell-cell variation was corrected for by regressing out differences in number of detected molecules, cell cycle state^57,58^ and percent mitochondrial gene content using the sctransform function^59^. Unsupervised principal component analysis, clustering, and non-linear dimensional reduction with UMAP was then used to detect the underlying cellular heterogeneity. Differential genes were identified using the FindAllMarkers function which uses a non-parameteric Wilcoxon rank sum test. Cluster identities were assigned through combination of immune ref using Single R and manual annotation. Differential gene expression analysis comparing two populations (ex. Vehicle vs eCNTFR) was performed using mixed modelling using MAST. The refractory to PD-1 gene list is shown in table 7 and refractory to PD-1 score was assessed by scoring each cell for depression of the signature using the AddModuleScore function. Enrichment/depletion of cell clusters compared to vehicle treated tissue was determined using the Pearson residuals as per Pelka et al^60^.

### NanoString.

Formalin-fixed, paraffin-embedded tissues were dewaxed in CitriSolv (no. 1601, DeCon), then rehydrated in two washes each of 100% ethanol, 95% ethanol, and ddH20. Standard sodium citrate (pH 6.0) epitope retrieval was performed before washing in 1x TBS containing 0.1% Triton X-100 (no. TBST01-03, Bioland Chemicals). Samples were blocked in buffer W (no. 2-1003-100, Iba) for 1 hour at room temperature then incubated in primary antibodies overnight at 4 °C with UV-cleavable oligonucleotide-conjugated antibodies for DSP. The following day, nuclei were stained with a 1:10 dilution of SYTO 13 in 1x TBS-T for 15 minutes. Tissues were then incubated in 4% PFA for 30 minutes. Slides were then stained with the GeoMx solid tumor morphology kit (stains for DNA, PanCK and CD45) and oligo conjugated panels (Mouse Core Immune Cell Typing, Immune Cell Profiling and MAPK Modules), tumors were selected as regions of interest (ROIs). ROIs were then segmented into tumor or immune cell populations based of Cd45 positivity and oligos collected for digital counts were obtained for each target on the nCounter system.

### CLCF1 patient expression.

All TPM normalized counts for plotting gene expression are sourced from UCSC Treehouse (v11), representing a total of 12,499 samples from 49 different diseases.

### Cell Lines.

#### Conventional Culture.

All human cell lines were obtained from ATCC and maintained in RPMI-1640 (no. 21870–076, Gibco) supplemented with 10% bovine growth serum (no. SH30541.03, Cytiva), 4mM glutamine and 1% penicillin and streptomycin (P/S) (10378–016, Gibco). Cells were incubated at 37° in 5% CO_2_. Cells were routinely STR (Promega GenPrint10), and Mycoplasma tested (ATCC Universal Mycoplasma Detection Kit).

#### Making Syngeneic Cell lines.

Cell lines were generated from mice collected at the 16-week timepoint as above. Mouse lungs were harvested, and tumors were macrodissected. Cells were dissociated as above and then plated into 10 cm dishes. Cell lines were grown and then reimplanted to ensure histology matched lung adenocarcinoma.

#### Creation of CNTFR knockout cell lines.

Human cell lines A549, H23, and H358 all were engineered to constitutively express Cas9 and then were transfected with sgRNA (Synthego). Briefly, 15 μL of 5 μM sgRNA in Opti-MEM (no. 31985–070, Gibco) was added to Lipofectamine^™^ RNAiMAX Transfection Reagent (no. 13778–150, Invitrogen) and incubated for 5 minutes. The transfection solution was then added to cells seeded the day prior. Media was changed after 24 hours. Single cell clones were created by performing a dual serial dilution starting with 10,000 cells. Colonies were allowed to form, then expanded and collected for verification of knockout. Knockout of CNTFR was verified using Inference of CRISPR Edits analysis (Synthego) on the sanger sequences.

### Cell Assays.

#### Cell Stimulations.

When cells reached 70–80% confluence they were serum starved for 16 hours in serum-free RPMI-1640. Cells were then stimulated with recombinant human CLCF1 (10nM, no. 962-CL, Biotechne) for 25 minutes. Cells were lysed and collected as previously described.

#### Cell Viability.

Cells were seeded in 96-well plates at 500 cells per well in a total volume of 100μl of media containing 10% BGS. Plates were imaged by Incucyte (Sartorious) every 8 hours for 7 to 10 days.

#### AMG510 Survival Curve.

Cells were seeded in 96-well plates at 2,500 cells per well 24 hours before treatment. Cells were treated with increasing concentrations of AMG510. Plates were imaged by Incucyte (Sartorious) every 8 hours for 7 days.

### Cell extracts and western blot analysis.

Cells were lysed in urea lysis buffer (50 mM Tris-HCl (pH 8.0), 150 NaCl, 8M Urea), 1× protease inhibitors (no. P8349–1ML, Sigma) and 1× phosphatase inhibitor cocktail (no. P5726–1ML, Sigma) for 15 min, then sonicated. Protein concentration was determined by BCA assay (Pierce). Samples were denatured by heat at 95°C for 10 min. Proteins were resolved by SDS–PAGE, transferred to a PVDF membrane and blocked in 5% non-fat dry milk. Primary antibodies were incubated overnight at 4 °C at the following concentrations: P-AKT (no. 9271, Cell Signaling, 1:750), T-AKT (no. 75692, Cell Signaling, 1:750), P-ERK1/2 (no. 4370, Cell Signaling, 1:1,000), T-ERK1/2 (no. 4695, Cell Signaling, 1:1,000), P-MEK1/2 (no. 9121, Cell Signaling, 1:1,000), T-MEK1/2 (no. 9122, Cell Signaling, 1:1,000), P-STAT3 (no. 9145, Cell Signaling, 1:1,000), T-STAT3 (no. 9139, Cell Signaling, 1:1,000), /β-Tubulin (no. 2148,Cell Signaling, 1:1,000), Actin (no. 3700, Cell Signaling, 1:1000). Proteins were imaged by ChemiDoc XRS System (Bio- Rad) and quantified using ImageJ software.

#### Cytokine Array.

Cytokine expression and kinase phosphorylation was measured in A549 with and without CNTFR knockout using the Proteome Profiler Human XL Cytokine Array Kit (no. ARY022B, R&D Systems) and the Proteome Profiler Human Phospho-Kinase Array Kit (no. ARY003C, R&D Systems). For both arrays, cells were serum starved for 16 hours, then stimulated with complete media containing 10% BGS. Cells for cytokine detection and kinase phosphorylation detection were stimulated for 8 hours and 25 minutes, respectively. Array procedure was carried out according to the manufacturer’s protocol. Proteins were imaged by ChemiDoc XRS System (Bio-Rad) and quantified using ImageJ software.

## References

[1] Vicent S (2012) Cross-Species Functional Analysis of Cancer-Associated Fibroblasts Identifies a Critical Role for CLCF1 and IL-6 in Non–Small Cell Lung Cancer In Vivo. Cancer Res 72:5744–5756 22962265 10.1158/0008-5472.CAN-12-1097 PMC3856949

[2] Song M (2021) Cancer-Associated Fibroblast‐Mediated Cellular Crosstalk Supports Hepatocellular Carcinoma Progression. Hepatology 73:1717–1735 33682185 10.1002/hep.31792

[3] Kim JW (2019) Antitumor activity of an engineered decoy receptor targeting CLCF1-CNTFR signaling in lung adenocarcinoma. Nat Med 25:1783–1795 31700175 10.1038/s41591-019-0612-2 PMC7087454

[4] Kwan AK, Piazza GA, Keeton AB, Leite CA (2022) The path to the clinic: a comprehensive review on direct KRASG12C inhibitors. J Exp Clin Cancer Res 41:27 10.1186/s13046-021-02225-w PMC8767686 35045886

[5] Lahiri A (2023) Lung cancer immunotherapy: progress, pitfalls, and promises. Mol Cancer 22:40 36810079 10.1186/s12943-023-01740-y PMC9942077

[6] Sharma P, Allison JP (2015) The future of immune checkpoint therapy. Science 348:56–61 25838373 10.1126/science.aaa8172

[7] Sahai E (2020) A framework for advancing our understanding of cancer-associated fibroblasts. Nat Rev Cancer 20:174–186 31980749 10.1038/s41568-019-0238-1 PMC7046529

[8] Propper DJ, Balkwill FR (2022) Harnessing cytokines and chemokines for cancer therapy. Nat Rev Clin Oncol 19:237–253 34997230 10.1038/s41571-021-00588-9

[9] Senaldi G (1999) Novel neurotrophin-1/B cell-stimulating factor-3: A cytokine of the IL-6 family. Proc. Natl. Acad. Sci. 96, 11458–11463 10500198 10.1073/pnas.96.20.11458 PMC18055

[10] Zhou Y (2023) Structural insights into the assembly of gp130 family cytokine signaling complexes. Sci Adv 9:eade4395 36930708 10.1126/sciadv.ade4395 PMC10022904

[11] Rose-John S (2018) Interleukin-6 Family Cytokines. Cold Spring Harb Perspect Biol 10:a028415 28620096 10.1101/cshperspect.a028415 PMC5793756

[12] Tuveson DA (2004) Endogenous oncogenic K-ras(G12D) stimulates proliferation and widespread neoplastic and developmental defects. Cancer Cell 5:375–387 15093544 10.1016/s1535-6108(04)00085-6

[13] Jackson EL (2005) The differential effects of mutant p53 alleles on advanced murine lung cancer. Cancer Res 65:10280–10288 16288016 10.1158/0008-5472.CAN-05-2193

[14] Menon D (2017) GSTO1–1 plays a pro-inflammatory role in models of inflammation, colitis and obesity. Sci Rep 7:17832 29259211 10.1038/s41598-017-17861-6 PMC5736720

[15] Zhu C (2022) Tumor microenvironment-related gene selenium-binding protein 1 (SELENBP1) is associated with immunotherapy efficacy and survival in colorectal cancer. BMC Gastroenterol 22:437 36253721 10.1186/s12876-022-02532-2 PMC9575293

[16] Ju X, Zhang H, Zhou Z, Wang Q (2019) Regulation of PD-L1 expression in cancer and clinical implications in immunotherapy. Am J cancer Res 10:1–11 PMC7017746 32064150

[17] Guo R (2019) Hypoxia-inducible factor-1α and nuclear factor‐κB play important roles in regulating programmed cell death ligand 1 expression by epidermal growth factor receptor mutants in non-small-cell lung cancer cells. Cancer Sci 110:1665–1675 30844110 10.1111/cas.13989 PMC6500984

[18] Kelly MR (2020) Combined Proteomic and Genetic Interaction Mapping Reveals New RAS Effector Pathways and Susceptibilities. Cancer Discov 10:1950–1967 32727735 10.1158/2159-8290.CD-19-1274 PMC7710624

[19] Sunaga N (2011) Knockdown of Oncogenic KRAS in Non–Small Cell Lung Cancers Suppresses Tumor Growth and Sensitizes Tumor Cells to Targeted Therapy. Mol Cancer Ther 10:336–346 21306997 10.1158/1535-7163.MCT-10-0750 PMC3061393

[20] Ostrem JM, Peters U, Sos ML, Wells JA, Shokat K, M. (2013) K-Ras(G12C) inhibitors allosterically control GTP affinity and effector interactions. Nature 503:548–551 24256730 10.1038/nature12796 PMC4274051

[21] Solanki HS (2021) Cell Type–specific Adaptive Signaling Responses to KRASG12C Inhibition. Clin Cancer Res 27:2533–2548 33619172 10.1158/1078-0432.CCR-20-3872 PMC9940280

[22] Araki K (2017) Translation is actively regulated during the differentiation of CD8 + effector T cells. Nat Immunol 18:1046–1057 28714979 10.1038/ni.3795 PMC5937989

[23] Verdon DJ, Mulazzani M, Jenkins MR (2020) Cellular and Molecular Mechanisms of CD8 + T Cell Differentiation, Dysfunction and Exhaustion. Int J Mol Sci 21:7357 33027962 10.3390/ijms21197357 PMC7582856

[24] Jiang W (2021) Exhausted CD8 + T Cells in the Tumor Immune Microenvironment: New Pathways to Therapy. Front Immunol 11:622509 33633741 10.3389/fimmu.2020.622509 PMC7902023

[25] Kamphorst AO (2017) Rescue of exhausted CD8 T cells by PD-1–targeted therapies is CD28-dependent. Science 355:1423–1427 28280249 10.1126/science.aaf0683 PMC5595217

[26] Masucci MT, Minopoli M, Carriero MV (2019) Tumor Associated Neutrophils. Their Role in Tumorigenesis, Metastasis, Prognosis and Therapy. Front Oncol 9:1146 31799175 10.3389/fonc.2019.01146 PMC6874146

[27] Miyake K (2023) Single-cell transcriptomics Identifies the differentiation trajectory from inflammatory monocytes to pro-resolving macrophages in skin allergy. 10.21203/rs.3.rs-2669348/v1 PMC10891131 38396021

[28] Li Y (2022) Occurrences and Functions of Ly6Chi and Ly6Clo Macrophages in Health and Disease. Front Immunol 13 10.3389/fimmu.2022.901672 PMC9189283 35707538

[29] Zahid KR (2022) Neutrophils: Musketeers against immunotherapy. Front Oncol 12:975981 36091114 10.3389/fonc.2022.975981 PMC9453237

[30] DuPage M, Dooley AL, Jacks T (2009) Conditional mouse lung cancer models using adenoviral or lentiviral delivery of Cre recombinase. Nat Protoc 4:1064–1072 19561589 10.1038/nprot.2009.95 PMC2757265

[31] Evans RA (2016) Lack of immunoediting in murine pancreatic cancer reversed with neoantigen. JCI Insight 1:e88328 27642636 10.1172/jci.insight.88328 PMC5026128

[32] McFadden DG (2016) Mutational landscape of EGFR-, MYC-, and Kras-driven genetically engineered mouse models of lung adenocarcinoma. Proceedings of the National Academy of Sciences 113, E6409–E6417 10.1073/pnas.1613601113 PMC5081629 27702896

[33] Adeegbe DO (2018) BET bromodomain inhibition cooperates with PD-1 blockade to facilitate antitumor response in Kras-mutant non-small cell lung cancer. Cancer Immunol Res 6, canimm.0077.2018 10.1158/2326-6066.CIR-18-0077 PMC6170698 30087114

[34] P rschke C (2016) Immunogenic Chemotherapy Sensitizes Tumors to Checkpoint Blockade Therapy. Immunity 44:343–354 26872698 10.1016/j.immuni.2015.11.024 PMC4758865

[35] Damme HV (2021) Therapeutic depletion of CCR8 + tumor-in ltrating regulatory T cells elicits antitumor immunity and synergizes with anti-PD-1 therapy. J Immunother Cancer 9:e001749 33589525 10.1136/jitc-2020-001749 PMC7887378

[36] Joshi NS (2015) Regulatory T Cells in Tumor-Associated Tertiary Lymphoid Structures Suppress Anti-tumor T Cell Responses. Immunity 43:579–590 26341400 10.1016/j.immuni.2015.08.006 PMC4826619

[37] Tay RE, Richardson EK, Toh HC (2021) Revisiting the role of CD4 + T cells in cancer immunotherapy—new insights into old paradigms. Cancer Gene Ther 28:5–17 32457487 10.1038/s41417-020-0183-x PMC7886651

[38] Shi Y (2022) Circulating cytokines associated with clinical outcomes in advanced non-small cell lung cancer patients who received chemoimmunotherapy. Thorac Cancer 13:219–227 34825500 10.1111/1759-7714.14248 PMC8758427

[39] Wang M (2021) The Role of Cytokines in Predicting the Response and Adverse Events Related to Immune Checkpoint Inhibitors. Front Immunol 12:670391 34367136 10.3389/fimmu.2021.670391 PMC8339552

[40] Iliopoulos D, Hirsch HA, Struhl K (2009) An Epigenetic Switch Involving NF-κB, Lin28, Let-7 MicroRNA, and IL6 Links Inflammation to Cell Transformation. Cell 139:693–706 19878981 10.1016/j.cell.2009.10.014 PMC2783826

[41] Iliopoulos D, Jaeger SA, Hirsch HA, Bulyk ML, Struhl K (2010) STAT3 Activation of miR-21 and miR-181b-1 via PTEN and CYLD Are Part of the Epigenetic Switch Linking Inflammation to Cancer. Mol Cell 39:493–506 20797623 10.1016/j.molcel.2010.07.023 PMC2929389

[42] Wei D (2003) Stat3 activation regulates the expression of vascular endothelial growth factor and human pancreatic cancer angiogenesis and metastasis. Oncogene 22:319–329 12545153 10.1038/sj.onc.1206122

[43] Voron T (2015) VEGF-A modulates expression of inhibitory checkpoints on CD8 + T cells in tumors. J Exp Med 212:139–148 25601652 10.1084/jem.20140559 PMC4322048

[44] Zhao Y (2022) VEGF/VEGFR-Targeted Therapy and Immunotherapy in Non-small Cell Lung Cancer: Targeting the Tumor Microenvironment. Int J Biol Sci 18:3845–3858 35813484 10.7150/ijbs.70958 PMC9254480

[45] Wu X (2017) Angiopoietin-2 as a Biomarker and Target for Immune Checkpoint Therapy. Cancer Immunol Res 5:17–28 28003187 10.1158/2326-6066.CIR-16-0206 PMC5215959

[46] Xuan Z-X, Zhang S, Yuan S-J, Wang W, Yu J (2016) Prognostic value of angiopoietin-2 in non-small cell lung cancer patients: a meta-analysis. World J Surg Oncol 14:237 27589869 10.1186/s12957-016-0992-4 PMC5010677

[47] Zhou S-L (2016) Tumor-Associated Neutrophils Recruit Macrophages and T-Regulatory Cells to Promote Progression of Hepatocellular Carcinoma and Resistance to Sorafenib. Gastroenterology 150:1646–1658e17 26924089 10.1053/j.gastro.2016.02.040

[48] Zhang Z (2020) The miR-30a-5p/CLCF1 axis regulates sorafenib resistance and aerobic glycolysis in hepatocellular carcinoma. Cell Death Dis 11:902 33097691 10.1038/s41419-020-03123-3 PMC7584607

[49] Pascual-García M (2019) LIF regulates CXCL9 in tumor-associated macrophages and prevents CD8 + T cell tumor-infiltration impairing anti-PD1 therapy. Nat Commun 10:2416 31186412 10.1038/s41467-019-10369-9 PMC6559950

[50] Hunter SA (2021) An engineered ligand trap inhibits leukemia inhibitory factor as pancreatic cancer treatment strategy. Commun Biol 4:452 33846527 10.1038/s42003-021-01928-2 PMC8041770

[51] Johnson DE, O’Keefe RA, Grandis JR (2018) Targeting the IL-6/JAK/STAT3 signalling axis in cancer. Nat Rev Clin Oncol 15:234–248 29405201 10.1038/nrclinonc.2018.8 PMC5858971

[52] Choy EH (2020) Translating IL-6 biology into effective treatments. Nat Rev Rheumatol 16:335–345 32327746 10.1038/s41584-020-0419-z PMC7178926

[53] Jackson EL (2001) Analysis of lung tumor initiation and progression using conditional expression of oncogenic K-ras. Genes Dev 15:3243–3248 11751630 10.1101/gad.943001 PMC312845

[54] Jonkers J (2001) Synergistic tumor suppressor activity of BRCA2 and p53 in a conditional mouse model for breast cancer. Nat Genet 29:418–425 11694875 10.1038/ng747

[55] LaFave LM (2020) Epigenomic State Transitions Characterize Tumor Progression in Mouse Lung Adenocarcinoma. Cancer Cell 38:1–31 32707078 10.1016/j.ccell.2020.06.006 PMC7641015

[56] Stuart T (2019) Comprehensive Integration of Single-Cell Data. Cell 177:1888–1902e21 31178118 10.1016/j.cell.2019.05.031 PMC6687398

[57] Barron L, Gharib SA, Duffield JS (2016) Lung Pericytes and Resident Fibroblasts Busy Multitaskers. Am J Pathol 186:2519–2531 27555112 10.1016/j.ajpath.2016.07.004 PMC5222977

[58] Buettner F (2015) Computational analysis of cell-to-cell heterogeneity in single-cell RNA-sequencing data reveals hidden subpopulations of cells. Nat Biotechnol 33:155–160 25599176 10.1038/nbt.3102

[59] Choudhary S, Satija R (2022) Comparison and evaluation of statistical error models for scRNA-seq. Genome Biol 23:27 35042561 10.1186/s13059-021-02584-9 PMC8764781

[60] Pelka K (2021) Spatially organized multicellular immune hubs in human colorectal cancer. Cell 184:4734–4752e20 34450029 10.1016/j.cell.2021.08.003 PMC8772395

